# The clinical potential of 1,5-anhydroglucitol as biomarker in diabetes mellitus

**DOI:** 10.3389/fendo.2024.1471577

**Published:** 2024-10-31

**Authors:** Haiying Xu, Renyin Chen, Xiaoli Hou, Na Li, Yanwei Han, Shaoping Ji

**Affiliations:** ^1^ Center of Molecular Medicine, Department of Basic Medicine, Shu-Qing Medical College, Zhengzhou, Henan, China; ^2^ Hospital Laboratory Department, Rehabilitation Hospital of Shu-Qing Medical College, Zhengzhou, Henan, China; ^3^ Department of Biochemistry and Molecular Biology, Medical School, Henan University, Kaifeng, Henan, China

**Keywords:** 1,5-anhydroglucitol, diabetes mellitus, biomarker, blood glucose, measurement

## Abstract

A crucial measure of diabetes management is to monitor blood glucose, which often requires continuous blood collection, leading to economic burden and discomfort. Blood glucose and glycated hemoglobin A1c serve as traditional indicators of glucose monitoring. But now glycated albumin, fructosamine, and 1,5-anhydroglucitol (1,5-AG) have been gaining more attention. 1,5-AG is a chemically stable monosaccharide that exists in the human body. Its serum concentration remains stable when blood glucose levels are normal. However, it decreases when blood glucose exceeds the renal glucose threshold. Studies have shown that 1.5-AG reflects blood glucose changes in 1 to 2 weeks; therefore, decreased levels of serum 1,5-AG can serve as a clinical indicator of short-term blood glucose disturbances. Recent studies have shown that 1,5-AG can be used not only for the screening and managing of diabetes but also for predicting diabetes-related adverse events and islet β cell function in prediabetic patients. In addition, saliva 1,5-AG demonstrates potential value in the screening and diagnosis of diabetes. This review focuses on the biological characteristics, detection methods, and clinical application of 1,5-AG to promote understanding and applicable research of 1,5-AG in the future.

## 1 Introduction

Diabetes mellitus (DM) is a chronic and systemic metabolic disease characterized by high blood glucose levels resulting from insufficient insulin secretion or insulin resistance. It includes prediabetes, type 1 diabetes mellitus (T1DM), type 2 diabetes mellitus (T2DM), gestational diabetes mellitus (GDM), and other special types of diabetes. Chronic hyperglycemia and glycemic fluctuations can lead to the development of complications such as cardiovascular disease, kidney disease, and retinopathy, which pose serious health risks (1, 2). According to the latest report by the International Diabetes Federation, the number of adults with diabetes has reached 463 million worldwide, and the prevalence rate of diabetes is as high as 9.3% (3).

Early diagnosis, regular screening, and long-term management of diabetes are crucial for controlling the condition. DM is typically diagnosed by assessing fasting blood glucose (FBG), postprandial blood glucose (PBG), random blood sugar (RBG), or 2-hour postprandial blood glucose(2hPG) levels. Monitoring methods include capillary blood glucose monitoring, continuous blood glucose monitoring, and glycosylated hemoglobin A1c (HbA1c) measurement, which is essential for evaluating long-term glycemic control (4, 5). In recent years, nontraditional glycaemia markers such as fructosamine, glycated albumin (GA), and 1,5-anhydroglucitol (1,5-AG) have gained more attention. HbA1c reflects average glucose levels over 2 to 3 months, while fructosamine and GA indicate changes over 2 to 3 weeks, which are influenced by variations in red blood cells and albumin levels, thus necessitating simultaneous glycemia monitoring (6, 7).

1,5-AG is gaining attention for its effectiveness in blood glucose monitoring. It is a glucose analogue that is ingested through food and excreted by the kidney. Serum 1,5-AG levels decrease when urinary glucose exceeds the renal threshold, reflecting onset of hyperglycemia, but gradually normalize as blood glucose levels return to normal levels (8).Unlike HbA1c,FA,and GA,1,5-AG provides insights into average blood glucose, postprandial hyperglycemia, and blood glucose variability in 1-2 weeks (6, 7). In 2003, the FDA approved the Glyco-Mark kit for detecting serum 1,5-AG, establishing it as a new tool for short-term glucose monitoring (9). In 2015, the Chinese Guidelines for the Clinical Application of Blood Glucose Monitoring proposed 1,5-AG as a new adjunctive indicator for blood glucose monitoring (10). Researches on 1,5-AG in screening,management,and risk assessment of diabetes have been expanding. Furthermore, salivary 1,5-AG has been explored as a noninvasive index for screening and diagnosis of diabetes. This review summarizes the progress of clinical application of 1,5-AG in diabetes to enhance further research.

## 2 Metabolism and effect of 1,5-anhydroglucitol

### 2.1 Structure and discovery of 1,5-anhydroglucitol

**Figure 1:**
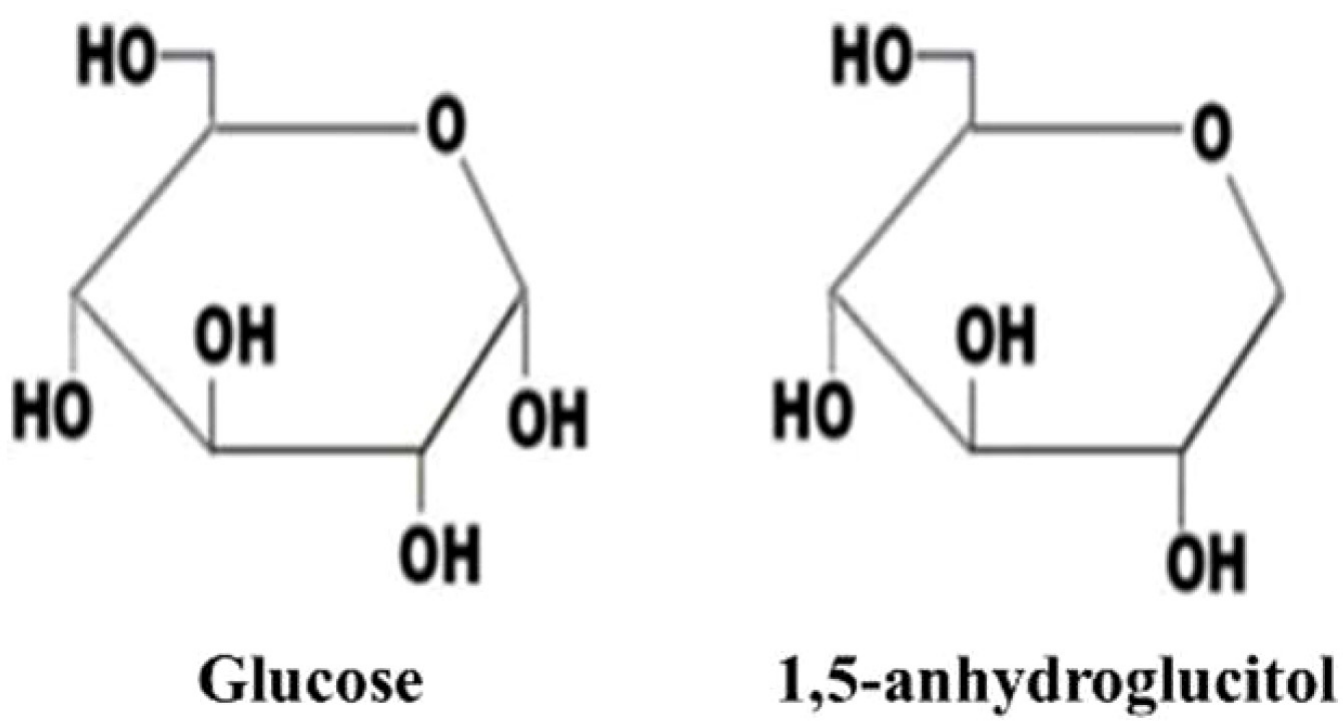
molecular structure of glucose and 1,5-Anhydroglucitol.

1,5-AG is a structurally similar but non-metabolizable analog of glucose. It is a six-carbon chain monosaccharide, C6H12O5 (164.2), lacking the hydroxyl group at the C-1 position (11, 12). Refer to **Figure 1** for its structure and physical properties. The closed pyranose ring structure maintains the stability of its chemical properties (13).

In 1888, the compound known as 1,5-AG was initially identified within the Polygala Senega plant group (14). Its molecular configuration was elucidated in 1943 (11). 1,5-AG is found in various foods, with soybeans being the primary source, and small amounts present in rice, pasta, fish, fruits, vegetables, tea, and milk (13). The presence of 1,5-AG in human blood and spinal fluid was confirmed in 1972 and 1973 (15, 16).

### 2.2 Dietary sources and metabolism of 1,5-anhydroglucitol

Blood 1,5-AG primarily comes from dietary sources rich in 1,5-AG and is absorbed by the intestines at a rate of about 4.38 mg/d (13). It is present in all human organs and tissues in a free form, with significantly higher quantities than in the plasma (6, 7). Studies in rats have suggested that the total amount of 1,5-AG in the human body ranges from 500 to 1000 mg (17). The typical reference range for 1,5-AG in humans is approximately 12-40 μg/mL, ranking second most prevalent polyol after glucose (18).

M. Suzuki et al. validated the synthesis of 1,5-AG in a rat hepatoma cell line, Reuber H-35, demonstrating that 1,5-AG was derived from glucose with an intermediate in its production (19). Subsequently, They identified 1,5-anhydrofructose(1,5-AF) as the immediate precursor in erythroleukemia cells (K-562), suggesting that 1,5-AG originated from glycogen in mammals due to its role as the precursor of 1,5-AF (20). Ying. L. et al. confirmed that 1,5-AG could move freely in and out of cells to maintain a dynamic balance in HepG2, C2C12, and primary mouse hepatocytes when incubated with 1,5-AG (18). Furthermore, administering 1,5-AF to Micromini pigs orally or intravenously, or to human subjects orally, resulted in the conversion of 1,5-AF to 1,5-AG in both human and swine (21).

After oral ingestion, 1,5-AG is efficiently absorbed in the intestine, distributed throughout the body, and excreted by the kidneys. It passes through the glomeruli and is mostly reabsorbed in the renal tubule by specific sodium glucose-linked cotransporters (SGLTs), maintaining a stable blood concentration of 1,5-AG through a balance of intake and urinary excretion (13, 22). Recent research has shown that the type and amount of dietary carbohydrates can influence 1,5-AG levels in non-diabetic adults, challenging the notion that 1,5-AG levels remain constant without hyperglycemic spikes (23). SGLTs, particularly SGLT4 and SGLT5, play a vital role in sugar absorption in both small intestine and renal tubules (22, 24, 25).

The tubular reabsorption of 1,5-AG competes with glucose. When blood glucose exceeds the renal threshold, glucose hinders the reabsorption of 1,5-AG, leading to increased excretion in urine and decreased serum levels (26). Serum 1,5-AG levels recovered at a rate of 0.3 µg/ml per day under tight glycemic control (9).Thus,1.5-AG reacts promptly and sensitively to fluctuations in blood glucose, indicating increase the glycaemia levels between 48 hours and 2 weeks (27).

### 2.3 Physiological effect on body

A study in rats and humans have showed that 1,5-AG inhibits disaccharidases such as trehalose, lactase, sucrase, and maltase. This inhibition reduces blood glucose and insulin levels by blocking glucose absorption and sucrase activity when 1,5-AG is ingested simultaneously with glucose (28). Furthermore, Genome-wide correlation studies have shown that genes associated with 1,5-AG-linked loci influence carbohydrate digestion and glucose transport, thereby impacting glucose levels through glucose metabolism (29).

Yamanouchi et al. investigated the impact of 1,5-AG on insulin secretion and found that 1,5-AG increased insulin secretion in RINr and MIN6 cells in a dose-dependent manner through a mechanism distinct from glucose, showing an additive effect with other saccharides and polyols (30).However, organ bath experiments revealed that 1,5-AG did not enhance insulin secretion in vivo and *in vitro* studies using isolated rat pancreas (31, 32).

T2DM patients are vulnerable to lipopolysaccharides (LPS), which stimulate macrophages to produce high levels of TNF-α and IL-6. In a research to determine 1,5-AG’s effect on inflammation, db/db mice and RAW264.7 cells were pretreated with 1,5-AG before exposure to a LPS challenge. The study found that1,5-AG reduced the cytokines release and protected db/db mice from pulmonary inflammation induced by LPS, by inhibiting Akt/NF-kB activity to decrease cytokine release and iNOS expression (33).

## 3 Measurement of 1,5-anhydroglucitol

1,5-AG can be determined in various samples such as serum, plasma, urine, saliva, vitreous humor, and cerebrospinal fluid. 1,5-AG detection methods include liquid or gas chromatography (LC/GC), mass spectrometry (MS), enzymatic methods, and enzyme-linked immunosorbent assay (ELISA). LC and MS are sensitive and precise but cumbersome and high cost (34). There are two enzyme assays for determining 1,5-AG in the blood, including GlycoMark assay (GlycoMark, Inc) approved in USA and Determiner-L (Kyowa Medex, Tokyo) used in Japan. These assays can be utilized interchangeably, though they may yield different results for the same samples (35). In recent years, researchers have developed some new methods, such as liquid chromatography-mass spectrometry (LC-MC), enzyme-based sensors and electrochemical biosensor, providing a possibility for detection of 1,5-AG in various samples (36–39). Saliva 1,5-AG has been successfully detected using both enzymatic and LC-MS methods. The levels of 1,5-AG in saliva detected by LC-MS were well consistent with those found in serum or saliva using enzymatic method. However, the consistency between saliva 1,5-AG levels detected by enzymatic methods and those in serum detected by the same method was lower due to interference from pyranose in saliva (40).

It is important to note that the reference intervals for serum 1,5-AG vary by age and sex. For males and females, respectively, the reference intervals were as follows: children, 96-302 and 89-277 μmol/L; adolescents, 84-311 and 79-277 μmol/L; and adults, 80-260 and 62-241 μmol/L (41). A study of nearly 1,800 health populations in the United States definitively established that the reference range of 1,5-AG is 8.4-28.7 μg/mL (42). Chen et al. found that the reference interval for 1,5-AG is 15.8~52.6 μg/mL for males and 14.3-48.0 μg/mL for females in Jiangsu Province (43). Jian C.H. et al. measured subjects with normal glucose tolerance and discovered that saliva 1,5-AG levels were 0.53(0.35-0.77)μg/mL(n=224) by LC-MS method and 3.30(2.30-4.30)μg/mL(n=175) by enzymatic method. The normal reference interval for saliva 1,5-AG levels is 0.086 to 1.627 μg/mL, as measured by the LC-MS method (40).

## 4 Serum 1,5-anhydroglucitol and diabetes mellitus

### 4.1 1,5-AG and screening of diabetes

The early detection of diabetes is crucial for effective prevention and treatment. Currently, FPG is a more commonly used indicator for clinical diabetes screening. However, FPG alone may miss patients with isolated postprandial hyperglycemia. Subsequent studies have demonstrated that 1,5-AG reflects the average blood glucose level over the past 1–2 weeks and can serve as an additional indicator for more comprehensive diabetes screening (6).

In Chinese individuals at high risk of diabetes, the mean 1,5-AG level in diabetic participants was significantly lower than in non-diabetic participants and shown a negative associated with FBG, PBG and HbA1c (44, 45).Serum 1,5-AG could be an effective indicator for diabetes screening with a cutoff of 11.18 μg/ml, a sensitivity of 92.6% and a specificity of 82.3% (44). Compared with FPG alone, the combination of 1,5-AG and FPG significantly increased the sensitivity of diabetes detection to 97.1% (46).The combination of 1,5-AG and FPG allowed 75.8% of participants to skip the OGTT, improving the efficiency of diabetes screening with a sensitivity of 82.5% and a specificity of 83.5% (45). 1,5-AG did not correlate well with 2hPG and showed high clinical specificity but low sensitivity for detecting hyperglycemia (47). Similarly, combining HbA1c with 1,5-AG may increase the sensitivity of diabetes screening, and decrease the rate of missed diagnoses and reduce the need for OGTT testing. In addition, 1,5-AG has advantages in individuals with mild glucose metabolic abnormalities such as hyperuricemia (48). In a Chinese population with normal glucose tolerance, serum 1,5-AG levels were lower in first-degree relatives of individuals with diabetes (FDR) than in non-FDRs, and were more sensitive to early glucose metabolism disturbance than HbA1c or GA levels (49).

T1DM is an autoimmune disease predominantly affecting young white people, characterized by more persistent hypoglycemia and severe glycemic excursions compared to T2DM. The serum 1,5-AG levels were significantly lower in both adults and children with T1DM than in those without T1DM. Therefore, serum 1,5-AG level may serve as an adjunct measure of hyperglycemia and a potential marker for diagnosing and screening of T1DM (50). Fulminant type 1 diabetes mellitus (FT1DM) occurs abruptly and usually manifests within one week after the onset of hyperglycemic symptoms.1,5-AG/GA index (AGI) can assist in the early differentiation between FT1DM and T1DM when HbA1c levels are below 8.7%, with an ideal threshold of 0.3, making it an applicable marker for early FT1DM identification (51).

GDM is a common metabolic disturbance during pregnancy. It was found that some differentially expressed metabolites, including 1,5-AG, were associated with the risk of GDM (52). A study found that average levels of 1,5 AG were notably reduced in women with GDM compared to those without GDM. 1,5 AG may identify GDM with a cut-off of 13.21 μg/mL, showing a sensitivity of 67.6% and a specificity of 65.3%. These results suggest that 1,5-AG may also be a marker for the GDM (53).

### 4.2 1,5-AG and glycemic control of diabetes

Good glycemic control is the main strategy for preventing the occurrence and progression of diabetic chronic complications. 1,5-AG has been proposed as a marker for short-term glycemic control and postprandial hyperglycemia.

Serum 1,5-AG level was significantly related to the glycemic excursion in subjects with T1DM. The 1,5-AG levels in children with T1DM were lower compared to normal controls and negatively associated with the peak post-meal glucose levels, supporting the potential of 1,5-AG levels to predict the postprandial hyperglycemia in managed T1DM children (54). Adolescents and young adults with T1DM have similar 1,5-AG levels that were lower than controls, which indicated that 1,5-AG could be used in the assessment of glycemic control in young patients with T1DM and HbA1c<8% (55). Thus, 1,5-AG may be used to assess short-term glycemic variability and may have a clinical relevance for monitoring glycemic excursions in T1DM (56).

1,5-AG has been proved to be a more effective indicator of glycemic fluctuations in T2DM patients compared to HbA1c and GA, and particularly effective measure for evaluating postprandial glucose in patients with moderate and well-managed conditions (57, 58). Unlike HbA1c, which may not accurately reflect the glycemic control in patients with hemolysis, 1,5-AG and GA are not influenced by hemolysis, and provide a precise measure of the glycemic control (59). The clinical trials have found that baseline 1,5-AG had an impact on the treatment effect of basal-bolus therapy, indicating that T2DM patients with lower 1,5-AG may experience greater benefit compared to patients with higher 1,5-AG (60).

Sodium–glucose cotransporter 2 inhibitors (SGLT2i) lower blood glucose by inhibiting the glucose reabsorption in the renal tubules and increasing urinary glucose excretion. Treatment with SGLT2 inhibitors lowered the HbA1c levels, with a more pronounced effect observed in patients with low 1,5-AG level. Blood 1,5-AG levels were lower in SGLT2i users. Based on these results, 1,5-AG may be as a useful marker of SGLT2i use (61, 62). Furthermore, incorporating 1,5-AG into clinical care of DM may improve the quality of care provided by primary care physicians. Physicians in the standard care plus 1,5-AG group were better able to identify timely patients with poor glycemic control and improve the quality of care. These findings support the clinical benefit of monitoring 1,5-AG in the management of diabetes (63).

### 4.3 1,5-AG and diabetic complications

When diabetes becomes uncontrolled, persistent hyperglycemia can damage blood vessels and vital organs to cause macrovascular complications (cardiovascular disease, cerebrovascular diseases) and microvascular complications (such as diabetic kidney disease, diabetic retinopathy) (64). Postprandial hyperglycemia and glycemic excursions have been shown to be associated with the onset of diabetic complications (65). Since serum 1,5-AG reflects hyperglycemia over the previous 1-2 weeks, many studies have investigated the correlations between serum 1,5-AG level and diabetic complications. Warren et al. followed 6644 participants without diabetes for ∼20 years and found that serum 1,5-AG level below 10 µg/mL was significantly associated with incidence diabetes and adverse outcomes compared to 1,5-AG level ≥10 µg/mL. This finding establishes a clear risk threshold for 1,5-AG levels <10 µg/mL (66).

#### 4.3.1 Diabetic cardiovascular and cerebrovascular diseases

Many studies have shown that decreased serum 1,5-AG levels are correlated with cardiovascular and cerebrovascular disease, making them an important indicator of the risk for Cerebral-cardio Vascular diseases in diabetic patients, including coronary heart disease, heart failure, chronic subclinical myocardial damage, acute coronary syndrome (ACS), and acute ischemic stroke (AIS)/transient ischemic attacks(TIA) (65, 67–71). For example, a reduced 1,5-AG level was strongly associated with coronary artery disease (CAD) and its severity in Chinese patients undergoing coronary angiography, and could be used to identify diabetes patients with significant glucose fluctuations and high risk of CAD in patients with diabetes (72). Additionally, in patients undergoing percutaneous coronary intervention (PCI), lower 1,5-AG level was independently associated with adverse clinical cardiovascular events (73, 74).

#### 4.3.2 Diabetic kidney disease

A comparative study of T2DM patients with stage I-III chronic kidney disease showed that serum 1,5-AG had a negative correlation with renal function compared to HbA1c and GA (75). Low 1,5-AG levels were correlated with higher risk of end-stage renal disease, independent of baseline renal function but not of glycemia (76). Another study evaluated the role of metabolites in predicting the primary outcome defined as need for dialysis, doubling of serum creatinine or death in Brazilian macroalbuminuric DKD patients. The results indicated that lower 1,5-AG levels were associated with development of macroalbuminuric DKD (77).

#### 4.3.3 Diabetic retinopathy

The relationship between 1,5-AG and diabetic retinopathy in patients with T2DM has been the subject of some research. It has been demonstrated that 1,5-AG levels are closely linked to diabetic retinopathy, particularly in patients with moderate glucose control, indicated by HbA(1c) levels below 8% (78). Furthermore, the ARIC study demonstrated that diabetic patients with low 1,5-AG levels (<6 μg/ml) had an 11-fold increased risk of retinopathy compared with diabetic patients with higher 1,5-AG levels (≥10 μg/ml), and this significance remained after adjusting for HbA1c and fasting blood glucose (79). Nevertheless, in a study of 2,681 community populations in Japan, Mukai et al. found that 1,5-AG was less sensitive than 2hPG in detecting diabetic retinopathy (80).

### 4.4 1,5-AG and islet β-cell function

In the prediabetic phase, the function of ß-cell mass diminishes to a crucial threshold, subsequently initiating diabetes. In a study by Won et al., individuals with lower levels of 1,5-AG exhibited higher insulin resistance and lower insulin secretion. Even in well-managed T2DM and prediabetes, reduced levels of 1,5-AG were strongly linked to reduced insulin secretion capacity (81).Likewise, in Chinese individuals recently diagnosed with T2DM, 1,5-AG was associated to basal insulin sensitivity and secretion, and strongly related to early-phase insulin secretion (82).

Furthermore, It was demonstrated that plasma 1,5-AG levels decreased in parallel with the loss of β-cells mass, revealing that 1,5-AG could reflect the drop of ß-cell mass in subjects at risk for diabetes, unlike other clinical parameters. Consequently,1,5-AG may serve as a biomarker of ß-cell mass in prediabetes (83).Based on their negative correlation and response to changes in glucose, Su Hang et al. proposed the AH index (AHI), which is calculated as 1,5-AG×HbA1c/100. They explored the possibility of AHI to be a new marker of glucose metabolism disorders and islet β-cell secretory function of T2DM patients. The findings indicated that the lower the AHI, the worse the glycemic fluctuation and the function of islet β-cell (84).

## 5 Salivary 1,5-anhydroglucitol and diabetes mellitus

Saliva is produced by the salivary glands, which are enriched with capillaries. Components from the bloodstream can penetrate the acinar and eventually be secreted into the saliva. Saliva is a complex fluid comprising water, proteins, metabolites, and microorganisms, making it a potential reservoir of biomarkers (85). Saliva biomarkers are considered functional equivalent to serum, as they partially reflect the physiological and pathological state of the body (86). Saliva is currently employed as a significant diagnostic fluid for the detection of many substances, including steroids, nonpeptide hormones, therapeutic drugs, and illicit drugs (87).

Saliva has recently attracted considerable interest in the search for disease biomarkers, offering an alternative to traditional diagnostic and screening methods due to its ease of collection, non-invasiveness, and improved patient compliance (88, 89). Currently, the non-invasiveness and simplicity of saliva have made it a focus for researchers investigating diabetes mellitus (86). A large number of studies on diabetic saliva biomarkers have been carried out at home and abroad, including salivary glucose, 1,5-AG, asprosin, resistin, and so on (86, 90–92).

Many studies have indicated that 1,5-AG can serve as a marker for the screening and diagnosis of diabetes (90). It was found that saliva 1,5-AG is significantly positively correlated with serum 1,5-AG and negatively correlated with blood glucose and HbA1c levels; therefore, it can be considered a reliable indicator for short-term glycemic control (93, 94). The GlycoMark assay of serum 1,5-AG showed a high correlation with the mass spectrometry (MS) measurements of serum 1,5-AG. However, the GlycoMark assay read-out of saliva 1,5-AG did not correlate with the MS measurements of saliva 1,5-AG. This discrepancy was due to the presence of galactose in saliva, which is analogous to 1,5-AG and affects the results of saliva assay readouts. Therefore, a modified 1,5-AG saliva analysis kit that involves enzymatically removing galactose is needed (95).

Furthermore, the combination of salivary 1,5-AG with FPG or HbA1c enhanced the effectiveness of screening for diabetes. Salivary 1,5-AG represented as a convenient and non-invasive method for screening diabetes (94). In diabetics, there is a significant negative correlation between postprandial blood sugar and 1,5-AG in both serum and saliva, which indicated 1,5-AG may serve as a valuable adjunct indicator for monitoring glycemic status in diabetic individuals (91). According to Ying, L.et al., salivary 1,5-AG was positively correlated with early-phase insulin secretion and notably positively associated with serum 1,5-AG, but negatively correlated with blood glucose markers (96). In a study conducted by Akito Sakanaka, salivary 1,5-AG and allantoin ranked in the top predictors of carotid atherosclerosis in T2DM patients undergoing treatment, and effectively discriminated those at high risk for CVD regardless of glycemic control status (97).

## 6 Conclusion and prospect

Diabetes mellitus is a significant threat to human health. Long-term fluctuations in blood glucose levels can cause the onset and progression of diabetic complications.1,5-AG, as a non-metabolizable analogue of glucose, has been confirmed to reflect hyperglycemia and fluctuations within a 1∼2 week period. In recent years, numerous studies have demonstrated that 1,5-AG can be utilized not only for screening, diagnosing, and managing diabetes but also for predicting and assessing its complications. Additionally, some studies have suggested that saliva can be utilized as a biological sample for diabetes-related research and clinical practice. Saliva 1,5-AG may serve as a potential marker for diabetes screening or surveillance. In summary,1,5-AG has shown great potential in all aspects of diabetes management and can be used as an additional index to traditional indicators. This could help establish a more comprehensive blood glucose monitoring system and thereby enhance the prevention and management of diabetes.

Although 1,5-AG has some clinical applications, there are still several issues that require further study. It is necessary to establish a standardized clinical reference range for 1,5-AG. Additionally, cumbersome and costly LC or MS methods need to be replaced with simpler and more convenient alternatives. Moreover, further research on the saliva 5-AG detection would be worthwhile. These studies will contribute to a more comprehensive of 1,5-AG as a marker for diabetes and enhance its application in the screening,diagnosis,surveillance,and prognosis of diabetes.
